# Free-electron laser data for multiple-particle fluctuation scattering analysis

**DOI:** 10.1038/sdata.2018.201

**Published:** 2018-10-02

**Authors:** Kanupriya Pande, Jeffrey J. Donatelli, Erik Malmerberg, Lutz Foucar, Billy K. Poon, Markus Sutter, Sabine Botha, Shibom Basu, R. Bruce Doak, Katerina Dörner, Sascha W. Epp, Lars Englert, Raimund Fromme, Elisabeth Hartmann, Robert Hartmann, Guenter Hauser, Johan Hattne, Ahmad Hosseinizadeh, Stephan Kassemeyer, Lukas Lomb, Sebastian F. Carron Montero, Andreas Menzel, Daniel Rolles, Artem Rudenko, Marvin M. Seibert, Raymond George Sierra, Peter Schwander, Abbas Ourmazd, Petra Fromme, Nicholas K. Sauter, Michael Bogan, John Bozek, Christoph Bostedt, Ilme Schlichting, Cheryl A. Kerfeld, Petrus H. Zwart

**Affiliations:** 1 Center for Advanced Mathematics in Energy Research Applications, Lawrence Berkeley National Laboratory, Berkeley, CA, USA; 2 Molecular Biophysics and Integrated Bio-imaging, Lawrence Berkeley National Laboratory, Berkeley, CA, USA; 3 Computational Research Division, Dept. of Mathematics, Lawrence Berkeley National Laboratory, Berkeley, CA, USA; 4 Hit Discovery, Discovery Sciences, IMED Biotech Unit, AstraZeneca, Gothenburg, Sweden; 5 Max-Planck-Institut für medizinische Forschung, Jahnstr. 29, 69120 Heidelberg, Germany; 6 Max Planck Advanced Study Group, Center for Free Electron Laser Science (CFEL), Notkestrasse 85, 22607 Hamburg, Germany; 7 University of Hamburg, Hamburg Germany; 8 Arizona State University, Tempe, AZ, USA; 9 Macromolecular Crystallography Group, Paul Scherrer Institute, 5232 Villigen – PSI, Switzerland; 10 European XFEL GmbH, Schenefeld, Germany; 11 Max-Planck-Institut für Kernphysik, Saupfercheckweg 1, 69117 Heidelberg, Germany; 12 Max Planck Institute for the Structure and Dynamics of Matter, Center for Free Electron Laser Science, Hamburg, Germany; 13 Max-Planck-Institut für extraterrestrische Physik, Giessenbachstrasse, 85741 Garching, Germany; 14 Carl von Ossietzky Universität Oldenburg, Department of Physics, Oldenburg, Germany; 15 PNSensor GmbH, Otto-Hahn-Ring 6, 81739 München, Germany; 16 University of California, Los Angeles, Los Angeles, CA, USA; 17 Department of Physics, University of Wisconsin-Milwaukee, 3135N. Maryland Ave, Milwaukee, WI 53211, USA; 18 Linac Coherent Light Source, SLAC National Accelerator Laboratory, Stanford, CA, USA; 19 Department of Physics, California Lutheran University, Thousand Oaks, CA, USA; 20 Laboratory for Macromolecules and Bioimaging, Paul Scherrer Institute, 5232 Villigen – PSI, Switzerland; 21 James R Macdonald Laboratory, Kansas State University, Manhattan, KS, USA; 22 Traction on Demand, Burnaby, BC, Canada; 23 Synchrotron SOLEIL, L’Orme des Merisiers, Saint-Aubin, BP 48, F-91192 Gif-sur-Yvette Cedex, France; 24 Department of Physics and Astronomy, Northwestern University, Evanston, IL, USA; 25 Atomic, Molecular and Optical Physics, Advanced Photon Source, Argonne National Laboratory, Argonne, IL, USA; 26 DOE Plant Research Laboratory, Department of Biochemistry and Molecular Biology, Michigan State University, East Lansing, MI, USA

**Keywords:** Computational biophysics, SAXS

## Abstract

Fluctuation X-ray scattering (FXS) is an emerging experimental technique in which solution scattering data are collected using X-ray exposures below rotational diffusion times, resulting in angularly anisotropic X-ray snapshots that provide several orders of magnitude more information than traditional solution scattering data. Such experiments can be performed using the ultrashort X-ray pulses provided by a free-electron laser source, allowing one to collect a large number of diffraction patterns in a relatively short time. Here, we describe a test data set for FXS, obtained at the Linac Coherent Light Source, consisting of close to 100 000 multi-particle diffraction patterns originating from approximately 50 to 200 *Paramecium Bursaria Chlorella* virus particles per snapshot. In addition to the raw data, a selection of high-quality pre-processed diffraction patterns and a reference SAXS profile are provided.

## Background & Summary

Fluctuation X-ray scattering (FXS) studies extend traditional small angle X-ray scattering (SAXS) methods by using X-ray snapshot with exposure times so short that the ensemble of illuminated particles can be well approximated as frozen in time and space. The resulting scattering patterns are no longer angularly isotropic, but instead exhibit small intensity fluctuations around the mean SAXS intensity^1,2^. Angular correlations of these intensity fluctuations can be directly related to the underlying molecular structure of the sample, providing much more information than traditional 1D SAXS curves^3^. The correlation function $C_{2}\left(q,q',\Delta \phi \right)$ is defined as

(1)C2(q,q′,Δϕ)=12πN∑j=1N∫02πIj(q,ϕ)Ij(q',ϕ+Δϕ)dϕ,

where N is the total number of diffraction patterns, *q* and *q*' are the magnitudes of the scattering vectors (in inverse resolution), and *ϕ* and *ϕ + *Δ*ϕ* are the corresponding angular coordinates describing the intensity *I*_*j*_(*q*, *ϕ*) of the j^th^ scattering pattern recorded on the detector. The correlation function $C_{2}\left(q,q',\Delta \Delta \phi \right)$ can be written as a Legendre series:

(2)C2(q,q',Δϕ)=∑l=0,2,…∞klBl(q,q')Pl(cosθqcosθq'+sinθqsinθq'cosΔϕ),

where *P*_*l*_ is the Legendre polynomial of order *l* and $\theta_{q}=\arccos\left(q\lambda /4\pi \right)$, *λ* is the wavelength of the incident X-rays, and *k*_*i*_ is a scale factor equal to the number of particles in the beam for *l* > 0, and equal to its square for *l*=0. The expansion coefficients *B*_*l*_(*q*, *q*′) are in turn related to the spherical harmonic expansion coefficients *I*_*lm*_(*q*) of the 3D intensity scattering volume *I*(***q***) of the scattering particle, where ***q***=(*q*, *θ*, *ϕ*):

(3)Bl(q,q′)=∑m=−llI*lm(q)Ilm*(q′),

where, in polar coordinates,

(4)I(q,θ,ϕ)=∑l=0∞∑m=−llIlm(q)Ylm(θ,ϕ).

The intensity function is equal to the square of the modulus of the Fourier transform of the real-space object *ρ*(***r***) under investigation:

(5)I(q)=|ℱ[ρ(r)]|2,

where ℱ denotes the Fourier transform^1^.

Prior work in fluctuation scattering from biological samples has demonstrated that high-quality correlation data can be obtained from single-particle diffraction data^4^ and can be used for *ab initio* structure determination using the multi-tiered iterative phasing algorithm^5^. Although previous work has shown that the signal to noise ratio (SNR) of such data is independent of the number of particles per shot^5^ when the particles are in a vacuum, the relationship between number of particles and SNR in the presence of large buffer and detector backgrounds has not yet been studied. In this communication, we describe unprocessed, experimental multi-particle scattering data from which an FXS correlation data set can be derived. The data, obtained at the Atomic, Molecular and Optical (AMO) instrument at the Linac Coherent Light Source^6,7^, consist of close to 60 000 high quality scattering images of the *Paramecium bursaria Chlorella virus* 1 (PBCV-1, ~190 nm in diameter^8^) and 30 000 scattering images of the sample buffer. The images presented here provide the community with experimental data on which algorithms for processing fluctuation scattering data and structure solution can be tested. The data are deposited at the CXIDB^9^ in the form of hdf5 and xtc files.

## Methods

### Sample Preparation, Sample Delivery and Data collection

A batch of *Paramecium Bursaria Chlorella* virus 1 sample was prepared as described previously^10^. Here we used 1% triton instead of Nonidet and centrifuged the virus sample at 20,000 rpm in an ultracentrifuge. The pure virus sample was dialyzed against 50 mM 4-methylmorpholine^11^. The quality of the FXS data was gauged by comparing the derived SAXS data against a reference Small Angle Scattering curve obtained at the cSAXS beamline at the Swiss Light Source, at an energy of 11 keV. The sample used for FXS data collection was diluted with buffer to a concentration of approximately 5 × 10^11^ particles per ml.

**Figure 1:**
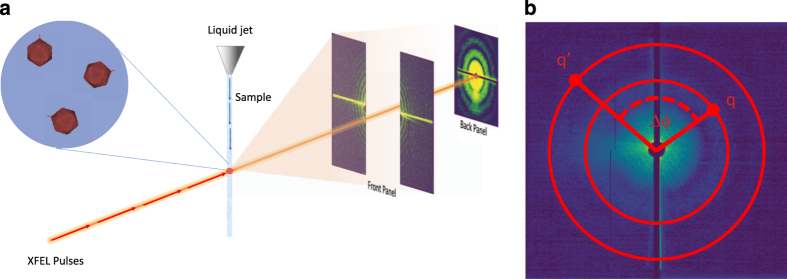
A schematic overview of a Fluctuation X-ray Scattering. (**a**) An FXS experiment at an XFEL can be performed by intersecting a liquid jet containing the sample particles with the XFEL pulse and recording the diffraction pattern on a multi-panel detector. The data generated by the setup described in this manuscript resulted in a maximum resolution of 14.3 nm (8.9 nm) at the edge (corner) of the front and 46.8 nm (32.7 nm) at the edge (corner) of the back detector. (**b**) Calculation of the intensity correlations is performed according to equation 1. The scattering patterns shown above are experimental data discussed in this report.

The multi-particle FXS scattering data were collected at the Atomic, Molecular and Optical (AMO) instrument at the LCLS^6,7^. The experiment was performed in the CFEL-ASG Multi-Purpose chamber (CAMP)^12^. The PBCV-1 solution described above was injected into the XFEL interaction region as a microjet of approximately 5 μm diameter, using a gas dynamic virtual nozzle (GDVN) injector^13,14^ at a flow rate of ~20 μl/min (Fig. 1). The diffraction data were collected in the water window, using a photon energy of 514 eV, an electron bunch length (pulse length) of 100 fs and a repetition rate of 120 Hz. The average number of photon per pulse was 10^12^. The focus size was approximately 25 μm^2^ (FWHM). Diffraction patterns were collected on two pairs of p-n junction charge-couple device (pnCCD) detectors^15^ read out at 120 Hz. The front and back panels of the detector consisted of two pairs of 1024 × 512 arrays of square 75 μm× 75 μm pixels. The front panels were placed 224 mm from the interaction region, separated by a horizontal gap of 23 mm. The back detector was placed 741 mm from the sample/XFEL interaction zone, with a horizontal gap of 1.73 mm (Table 1). The maximum resolution achievable under these conditions is 14.3 nm (8.9 nm) at the edge (corner) of the front and 46.8 nm (32.7 nm) at the edge (corner) of the back detector. The X-ray scattering patterns and associated metadata were stored as xtc files. These diffraction images were pre-processed using the CFEL-ASG Software Suite (CASS)^16^. The images were corrected for dark current; pixels systematically producing outlying intensity values were flagged. The resulting data was cast in larger arrays to include the detector gaps. The detector halves were placed roughly symmetrically around the X-ray beam. The back detector, with gaps included, is contained in an array of 1024 by 1047, with the mean beam center located at (506, 526). The front detector (with gaps included) is contained in an 1331 by 1031 array, with a mean beam center of (657, 552). The back detector was at gain mode 1, corresponding to 1250 ADU per 1keV photon. The gain mode of the front pnCCD was 4, corresponding to 78 ADU per 1keV photon. The resulting arrays are stored in an hdf5 file and are deposited in the CXIDB with accession number **79** (Data Citation 1). A Globus end-point is available for high-speed data-transfer.

From the concentration, the jet diameter and the focus size the particle count per exposure is estimated to 60 particles per shot. Given that the focussed X-ray beam has extended tails beyond the focal limit, there is an uncertainty that likely places the true particle count somewhat higher. A conservative estimate of the bounds of the particle count of 50 to 200 is proposed.

### Pattern Selection

**Figure 2:**
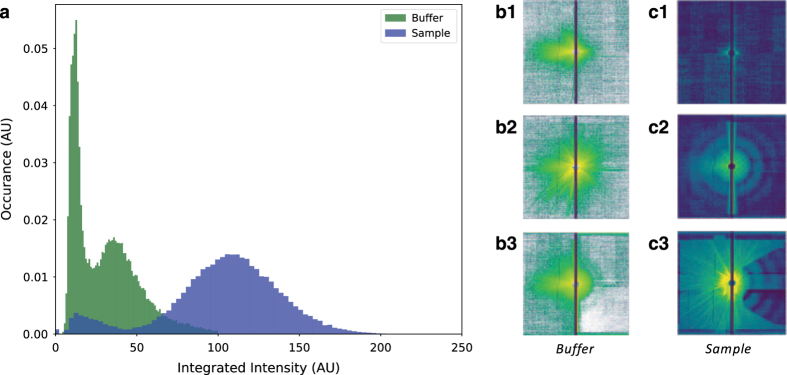
Pattern selection. (**a**). The total integrated intensities from the back panel form a bimodal distribution, for both the buffer data and the sample. In the buffer run, decent buffer-only shots (**b1**) dominate the low end, whereas streak dominated images (**b2**) and shots containing residual virus particles (**b3**) are found with higher integrated intensities. For the sample run, blank shots (**c1**) dominate the low end, and streak-dominated images (**c3**) are found in shots with integrated intensities residing in the extended tail (>200 AU) of the distribution. Diffraction patterns of the sample falling in the major peak (**c2**), with integrated intensities between 50 and 200 AU can be used to obtain experimental intensity correlations. All diffraction patterns are shown with a logarithmic colormap.

Due to liquid jet and X-ray beam instabilities, not all scattering patterns collected are of sufficient quality for correlation analyses. The set of diffractions patterns of the sample collected contain, besides multi-particle hits, a set of *blanks*, where no virus was intersected by the XFEL beam, as well as images characterized by a very high total scattered intensity and extensive intensity streaks where the X-rays hit the edge of the jet or part of the liquid-jet nozzle, Fig. 2. Similar observations were made for the buffer run. A selection of patterns was made on the basis of the total integrated intensity on the back panel. A histogram analysis of the integrated intensity reveals a bimodal distribution, with high quality patterns occurring around the most-populated mode of the distribution.

### Code Availability

CASS is publicly available on github (https://gitlab.gwdg.de/p.lfoucar/cass). The reading of xtc files is supported by the psana libraries distributed by the LCLS (https://stanford.io/2lhTEwT).

## Data Records

Four individual datasets have been deposited on the CXIDB website (Data Citation 1). The deposited data consists of the raw xtc file of the experimental PBCV-1 and buffer scattering data. Selected patterns, pre-processed with CASS, involving dark-current subtraction and common mode corrections, of PBCV-1 and buffer have been deposited also in separate hdf5 files for the back and front pnCCD detectors. The xtc files contain close to 100 000 scattering patterns, whereas the selected pre-processed files contain close to 60 000 patterns for PBCV-1 and 30 000 patterns for the buffer. A reference buffer subtracted SAXS data set from PBCV-1 at the same concentration, collected at 11 keV using the CSAXS beamline at the Swiss Light Source has been deposited as well. The data records are summarized in Table 2.

## Technical Validation

**Figure 3:**
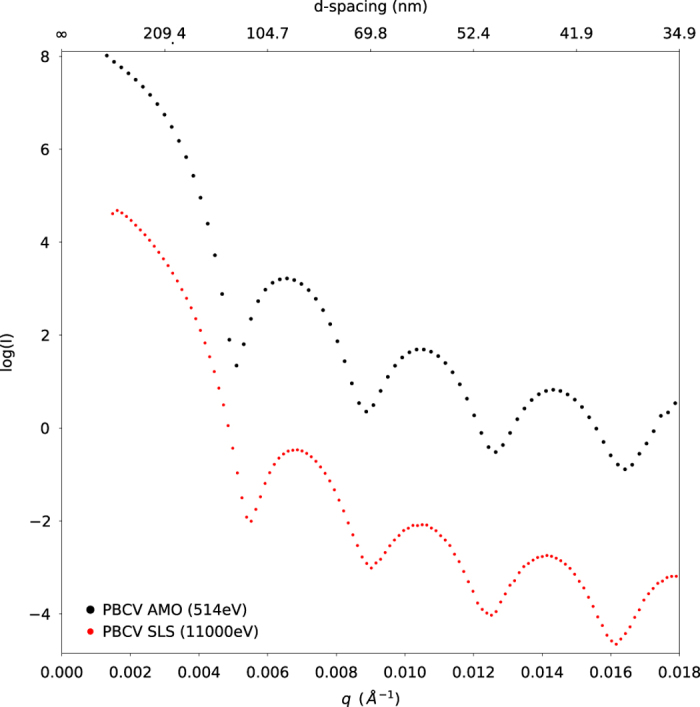
The experimental SAXS data derived from the selected snapshots on the back detector display a characteristic oscillatory behaviour consistent with spherical-like particles. This SAXS curve is close to a reference curve obtained from PBCV-1 at the Swiss Light Source’s CSAXS beamline at 11000 eV. Minor discrepancies between the soft X-ray and hard X-ray curve are likely due to contrast differences in the water window. A hard sphere model of the AMO data and the SLS data suggest a diameter of 174 nm and 168 nm, which would correspond to an icosahedron with diameter of 204 and 197 nm respectively.

The quality of the data can be assessed by the mean intensity as a function of resolution, Fig. 3. The SAXS curve was obtained by angular integration of the images after masking out the strong jet scattering streaks seen in the diffraction patterns, Fig. 2c. A mask covering the jet streak included in the deposited hdf5 files. The experimental SAXS data were fitted in the low-q region (up to 0.015 Å^−1^) with the theoretical scattering curve of a hard sphere with a diameter of 174 nm. Given that the diameter of an icosahedron is 17% larger than the sphere that touches the midpoint of each vertex^17^, the hard sphere model derived here would correspond to a maximum particle dimension of little over 200 nm, consistent with the available model^8^. The analyses of the reference data collected at the Swiss Light Source can be modelled (at low q) with a hard sphere with a radius of 168 nm, corresponding to an icosahedron with diameter of 197 nm. The difference in estimated size between the reference data and the curve obtained from AMO can be ascribed to changes in relative contrast at lower X-ray energies, the effects of radiation damage on the sample at synchrotron sources, variations in sample preparations or concentration effects on the shape of the low q data.

## Additional information

**How to cite this article:** Pande, K. *et al*. Free-electron laser data for multiple-particle fluctuation scattering analysis. *Sci. Data*. 5:180201 doi: 10.1038/sdata.2018.201 (2018).

**Publisher’s note:** Springer Nature remains neutral with regard to jurisdictional claims in published maps and institutional affiliations.

## Supplementary Material



## Figures and Tables

**Table 1:** Data collection parameters.

Parameter	Value
X-ray energy	**514 eV / 2.4 nm**
X-ray pulse length	100 fs
X-ray repetition rate	120 Hz
Front panel gap	23 mm
Front panel distance	224 mm
Back panel gap	1.73 mm
Back Panel distance	741 mm

**Table 2:** Data Records.

Data Identifier	Contents	Comments
CXIDB-79: Raw XTC Files	PBCV-1 and buffer scattering patterns in xtc format.	
CXIDB-79: PBCV Diffraction Patterns	Selected scattering images of PBCV-1 in solution, common mode and geometry corrected by CASS. Contains ~60000 patterns.	The hdf5 path ‘/data/data’ contains the data. The ‘/data/mask’ field contains a mask covering the overloaded jet streak. ‘data/timestamp_str’ contains timestamps of the images.
CXIDB-79: Buffer Diffraction Patterns	Selected scattering images of sample buffer, common mode and geometry corrected by CASS. Contains 20000 patterns.	The hdf5 path ‘/data/data’ contains the data. The ‘/data/mask’ field contains a mask covering the overloaded jet streak. ‘data/timestamp_str’ contains timestamps of the images.
CXIDB-79: SAXS Data	A reference SAXS curve of PBCV-1 collected at the cSAXS beamline at the Swiss Light Source, collected at an energy of 11keV.	A SAXS curve in plain ASCII. Each line contains q (in inverse Ångström) and the corresponding mean intensity.
